# Mind the Differences: How Diagnoses and Hospital Characteristics Influence Coordination in Cancer Patient Pathways

**DOI:** 10.3390/ijerph18168818

**Published:** 2021-08-21

**Authors:** Per Magnus Mæhle, Ingrid Kristine Small Hanto, Victoria Charlotte Simensen, Sigbjørn Smeland

**Affiliations:** 1 Department of Health and Society, Faculty of Medicine, University of Oslo, 0314 Oslo, Norway; 2 Comprehensive Cancer Centre, Division of Cancer Medicine, Oslo University Hospital, 0450 Oslo, Norway; insmal@ous-hf.no (I.K.S.H.); ssm@ous-hf.no (S.S.); 3 Department of Research Support-Clinical Trial Unit (CTU), Oslo University Hospital, 0424 Oslo, Norway; Victoria.C.Simensen@outlook.com; 4 Department of Clinical Medicine, Faculty of Medicine, University of Oslo, 0318 Oslo, Norway

**Keywords:** cancer patient pathways, integrated care pathways, cancer care, coordination, breast cancer, colorectal cancer, ovarian cancer

## Abstract

Integrated care pathway (ICP) is a prevailing concept in health care management including cancer care. Though substantial research has been conducted on ICPs knowledge is still deficient explaining how characteristics of diagnose, applied procedures, patient group and organizational context influence specific practicing of ICPs. We studied how coordination takes place in three cancer pathways in four Norwegian hospitals. We identified how core contextual variables of cancer pathways affect complexity and predictability of the performance of each pathway. Thus, we also point at differences in core preconditions for accomplishing coordination of the cancer pathways. In addition, the findings show that three different types of coordination dynamics are present in all three pathways to a divergent degree: programmed chains, consultative hubs and problem-solving webs. Pathway coordination also depends on hierarchical interaction. Lack of corresponding roles in the medical–professional and the administrative–institutional logics presents a challenge for coordination, both within and between hospitals. We recommend that further improvement of specific ICPs by paying attention to what should be standardized and what should be kept flexible, aligning semi-formal and formal structures to pathway processes and identify the professional cancer related background and management style required by the key-roles in pathway management.

## 1. Introduction

### 1.1. The Lack of Contextual Understanding in Cancer Patient Pathway Implementation

The integration of individual patient trajectories and the high flow of patients through hospitals are recognized as a major challenge in cancer care [1]. Integrated care pathways (ICPs), or cancer patient pathways (CPPs) as they are called in cancer care, were launched to address these challenges [2,3]. Such tools are associated with standardization, which in organizational research is recognized as a mechanism of coordination [4]. However, are CPPs capable of creating coherence, and can they be managed, across the silo-oriented hospital system? One would expect real-life pathway coordination to be influenced by what and who are treated by whom and where, to put it more precisely, by real-life diagnoses, patients and hospital organizational fields, and the multitude of directions and constraints these entail.

The scope [2,5,6] and purpose [5,7,8,9,10] of ICPs have been widely reported in the literature. Historically, the main driving force is the need to reduce the increasing tension between quality and cost-effectiveness of care [11]. ICPs are interpreted as a tool to implement clinical guidelines and evidence-based medicine [7,12] while monitoring medical practice and making it more accountable [13,14,15]. ICPs are also portrayed as a way of making health care more patient-centered and reducing variance in quality, cost and care [15]. ICPs were initially developed for other diagnoses than cancer. However, the significant contextual variations in cancer make this group of diagnoses well suited for investigating conditions for deploying ICPs.

Several definitions of ICP have been proposed, but there is no uniform or international standard defining what elements ICPs should contain or entail [16]. However, a core ingredient of the ICP phenomenon is the matrix of events or procedures along a timeline technically expressed through a flow chart or a Gantt chart [17,18]. An ICP is referred to as complex [19] as it entails several components in addition to the flowchart. In addition to a documented linear workflow process, introducing navigators or coordinators, multidisciplinary team meetings (MDT), patient information and education, and monitoring procedures all correspond with the primary objective for ICPs. Accordingly, ICPs are described as a method of governance, management, boundary processes enhancement or work process improvement.

Several scholars claim that the main purpose of ICPs is to improve coordination of care [18,20,21,22]. Coordination is a core activity in optimizing patient flow in hospitals. It is also essential in multi- and cross-disciplinary interactions and decision-making. Combining clinical and patient-related decisions and the logistics of cure and care is essentially coordination. The implementation of ICPs as a tool for coordinating activities and deployment of knowledge seems to unite the expressed motives and included measures. Using ICP as a coordination device is a solution to the divergence between increased fragmentation and the demand for integration. In a horizontal workflow, ICPs allow for coordination across formal organizational borders. Coordination also has a vertical dimension when the immediately involved health professionals are not authorized to make adjustments within the system or make necessary resources available. This is problematized in publications on ICP but remains unsolved [5,15].

While some authors seem to present ICPs as a kind of panacea for most problems in health care and hospitals [13], others point to the limitations of their effectiveness and validity of ICPs. Two widespread conclusions and recommendations on effectiveness and validity are as follows: Firstly, ICPs are best fit for high-volume diagnoses [3,21]. Secondly, ICPs work best in care processes with a high degree of predictability [8,20,23,24]. Some of the first reported clinics to organize ICPs were specialized orthopedic clinics [18]. Accordingly, the literature has debated the general usefulness of ICP as a tool to optimize care in more complex and/or less predictable patient pathways. Thus, one takeaway is that ICP is not a measure fit for achieving industrialized standardization when the conditions for such standardization are not present [25].

In the last 20 years, more complex pathways as in cancer care have widely incorporated ICPs or CPPs, and also in hospitals with relatively small volumes of patients and limited ability to create high process predictability. At an institutional level, low predictability is caused by variation in degrees of urgency, patient expectations and needs, and availability of resources. Many studies have described ICPs in practice or tried to evaluate their effects. However, so far literature point at some knowledge gaps [5,7,16,22,23,24]. First, reports are frequently limited to one hospital, one diagnose and pathway and one element of the pathway. Consequently, they lack a comprehensive perspective [7,26] since internal properties of a specific treatment or patient group [5] may influence the functioning of the CPP flow. Secondly, knowledge is missing on how different parts of CPPs work and in what contextual circumstances [7]. A CPP is more than a complex intervention. It is a complex intervention in a complex system [9]. This makes it challenging to analyze cause and effect processes [27], which leads to our research question: What traits of cancer diagnoses, patient characteristics and hospitals have a significant impact on cancer patient pathway coordination and how do these differences influence coordination processes and management requirements?

Several CPP studies deal primarily with existing approved ICP documents. Thus, the research is focused on the map rather than the mapping process and implementation based on the existence of some kind of pathway map [8,28]. In Norway, national standardized CPPs for all major cancer diagnoses were officially implemented in 2015 [29]. The main target of this reform was to improve flow time for patient throughput time from referral to start treatment [30]. However, in several hospitals in Norway, elements of CPPs were already present and had been put into practice. The national CPP documents gave room for customization [25]. What we will study is not primarily the intention of the CPPs as phrased in national documents, but real-life CPPs in hospitals in the context of cancer diagnosis, treatment, organizational structures and governance systems. In the current work, the term CPP refers to the pathway process as implemented while the term standardized CPP refers to officially approved, documented pathways.

### 1.2. Analytical Approaches to Explore Understand Crucial Differences between CPP Processes

We draw upon three analytical approaches to study different coordination mechanisms in CPPs. First, inspired by Trosman [1], we look at CPPs from a project management perspective. We assume that a project is a unique task with a definite end but will be carried out within some degree of uncertainty and complexity. From this, neither individual CPPs nor the total stream of diagnoses specific CPPs would per definition be a project if CPPs could be implemented in a standardized way based on predictable and satisfying access to resources and stable or predictable surroundings. Conversely, if both single CPPs and the total stream of CPPs have some degree of unpredictability and uncertainty [31] added by some extent complexity, they could be classified as series of project tasks constituting a program [32]. In line with Slack et al. [32] these CPP programs may be classified into a matrix in relation to the combined degree of uncertainty and the degree of complexity. Increased uncertainty in project-like tasks will lead to extended challenges to accomplish the up-front planning of the process. While increased complexity will challenge the ability to control the process while in progress. In identifying variable expressing uncertainty and complexity, we follow the analysis of Han et al. [31] proposing that a reasonable taxonomy of uncertainty in healthcare should be attached to the source from which it originates.

The project task principally cannot be solved in a satisfactory manner if all quality measurements, available resources and available time slots are fixed and do not leave room for any flexibility, slack and room for negotiation [32,33]. This challenge is exacerbated in situations characterized by extended complexity and shortage of available time limiting the ability to arrive at a complete overview of the chosen interventions and outcome [25,34]. This is presumably why ICPs primarily entered health care in sheltered elective pathways suited to deliver a predefined quality and volume with a fixed time frame and resource base [18,35]. If there is a temporary higher influx of patients in such cases, the patient can wait with hardly any clinical risk. It also explains the use of ICPs in acute settings such as trauma and stroke treatment. In such cases, time cannot be compromised. Neither can outcome quality. However, competent resources will be flexible and available when needed. These highly urgent pathways unfold impressively in lots of hospital acute care units and they are both documented and internalized among the potential participants. This raises several questions: How do we rate CPPs for different diagnoses in terms of complexity, variation and predictability of context and process? In addition, how flexible are they in terms of available time and perceived urgency and/or access to critical resources of equipment and competences? Quality of outcome can hardly be negotiated, but certain standard pathway processes should allow some room for improvisation to adapt to restricted flexibility of time and resources. This will open up for identifying the optimal way to implement CPPs under variable conditions [15,25] and provide knowledge on the type of and variation in complexity and uncertainty and thus open for identifying the corresponding management strategies. CPPs are interpreted as a measure to reduce complexity and unpredictability and thus make rational planning achievable [36] while others claim that these pathways have to adapt to the complexity and fluidity of the context in which they are deployed [7,37]. Answering our research question might also resolve this apparent contradiction.

The second analytical approach concerns searching for conceptual tools to differentiate between types of coordination dynamics in CPP processes. ICPs are explained as an organized and predefined flow of activities across a certain time span [28]. This is in line with the organizational model of the work chain [14]. However, we question whether this will cover all types of dynamics present in coordination of CPPs. Cancer care is increasingly complex. This is due to an increasing numbers of events and alternatives of procedures or treatment routes at each event [1,38]. Pathway activities include more steps requiring integrated decision-making and cross-disciplinary processes. In addition, the pathways are dynamic and tightly intertwined back-and-forth processes. New knowledge emerges in several steps and may change the route of the pathway. As a consequence logistics and knowledge interdependence of diagnostic and therapeutic activities become intrinsically interweaved. Concepts covering these types of process dynamics and appearing as alternatives to the programmed chain of activities have been described in the literature [39,40,41]. When studying hospital organization and clinical coordination, Glouberman and Mintzberg [41] add two models to the programmed chain: the consultative hub and the problem-solving web. In the consultative hub, one professional actor seeks assistance from other professionals with supplementary knowledge or skills. This first professional then has a coordinating role. In the problem-solving web, there is a cooperation among equals and all contributors are active coordinating partners. The concept of a web also has connotations of a network. In our study, we anticipate that the three concepts including programmed chain, consulting hub and problem-solving web are tools used fruitfully to explain variations in dynamics of CPP processes.

The third analytical approach, inspired by Greenwood et al. [42], includes the concept of institutional logics and the interplay between these in our analysis of vertical coordination. Several scholars have explored the concept of institutional logics or interests in relation to patient pathways [7]. The concept of institutional logic was introduced by Alford and Friedland [43]. They defined it as cultural beliefs and roles determining how practices and structures are assessed. Accordingly, we identify two dominant institutional logics present in hospitals: the professional medical logic based on a combination of scientific knowledge and experience-based skills in diagnostics and treatment, and the economic–administrative logic responsible for the optimal use of resources to deliver the outcome expected by the hospital owner under certain resource constraints. Thus, in line with other scholars [14,28], we do not view the ICP as an objective concept and practice that can be applied to every kind of interest and purpose involved in the pathway. Logics are in play, and they interfere with the specific unfolding of ICP, both documented as a map and in practice [38,44]. The professional medical logic defines the preconditions for horizontal coordination activities and is represented by the informal medical community of practice [45] and clinical guidelines [11]. The economic–administrative logic is present through the hierarchical processes of governance and is in touch with the ICP in conducting monitoring activities targeting the outcome parameters of ICP that has political and administrative attention like accomplishment of lead time in standardized CPPs. Furthermore, the economic–administrative logic meets the ICP when coordination at street-level raises question that needs to be elevated to ha higher organizational level to be solved; usually lack of resources or adjustments to supportive systems [46]. The structuring of interaction between organizational levels affects the ability to achieve balanced solutions to coordination challenges when premises from two different institutional logics are present [7,13,44,46]. In this study, we search for traits of cancer diagnoses and hospitals, including relations between hospitals that have an impact on these interaction processes and thus might constitute decisive differences in the way each CPP works.

The three analytical approaches identifying crucial contextual variables making an impact on coordination of CPPs also relates to differences in management. This is a main point of Buchanan et al. [47] in a study of change management for the prostate cancer pathway in British hospitals. They suggest that the content of process and the degree of complexity of influence which leadership style that are contributing to success. Increased complexity and a process associated with ambiguity and blurred borders correspond to a need of greater flexibility supported by enhancing distributed leadership. The connection between the type of context-dynamics, management roles and leadership styles are elaborated in management literature. Moreover, further analysis of managing different CPP under various circumstances could demand both roles as controller [48], integrator [49], broker and steward [50]. Thus, they provide us with a potential conceptual tool to speculate on the connection between variations in context and requirements for specific managerial roles.

## 2. Materials and Methods

CPPs consist of several unique independent elements that are constructed in various ways, which, separately and in combination, may influence the outcome. In addition, the field of interest has several elements that might individually and in combination, directly or indirectly, affect outcomes. In the research design, we also have to consider that the field context is not stable but dynamic and non-linear. Since experimental methods are not suitable to studying CPPs as a complex intervention in a complex system [20,51], we approached the epistemological puzzle created by the several layers of complexity, instability and iterative processes on one hand by using a research design comprising two elements: multi methods and combination of data sources [51,52,53]. We allowed theory to emerge from the field [54] and being fertilized by diverse models from previous research in a theoretical triangulation [53]. The theoretical models were identified through an abductive process while structuring the data from our cases [55].

When searching for variables that are decisive for CPP execution, we selected case signals and underlying hypothesis regarding variables that might have an explanatory value. We chose to investigate the pathways of three cancer diagnoses. In selecting colorectal cancer, breast cancer and ovarian cancer, we had pathways that differed in terms of patient volume, degree of urgency, existence of screening programs, proportions of patients receiving multimodal therapy, referral patterns to university hospitals, and whether the surgical activity is sheltered from emergency activity. By selecting both university hospitals and community hospitals, we captured differences in size and variations in the proportion of specialized care. Choosing two hospitals in each group allowed us to evaluate how the same role in cancer care could be accomplished in different ways with possible impact on CPP execution. The four hospitals participating in the study, two university hospitals and two community hospitals, represent two health regions. The Norwegian hospitals are organized in four health regions governed by a governmentally owned regional health trust. There is one referral hospital in each region and a regional referral plan centralized treatment. This implies that the university hospitals act as a regional hub for specialized care.

The main sources of data were qualitative interviews and documents. Relevant documents were documents from the hospitals’ quality systems, including procedures for practicing CPPs; information available on the hospital website, like organizational maps and relevant policy documents; data from the national CPP monitoring system; national diagnosis-specific guidelines approved by the Directorate of Health; and statistics from the National Cancer Registry and the national diagnosis-specific quality registers. We identified core formal sources of relevant documents based on information from our contact persons, during interviews, and from the authors’ knowledge in the field.

In each hospital, we had a contact person who gave us information about the hospital, procured relevant documents, and identified relevant interviewees. We picked the informants to represent all key activities for all three pathways at every hospitals. This means key medical personnel from outpatient units, surgery, oncology, pathology and radiology departments. Some were leaders; others had no formal management position. In addition, we interviewed patient coordinators, the majority of whom were trained nurses. We also interviewed some department leaders. Some leaders were responsible for more than one of the diagnoses and CPPs; this was more common in the community hospitals. Except for two interviews, all of the 66 interviews were performed in the interviewee’s local environment. A loose interview guide was distributed to the interviewees ahead of the interview. The interviews lasted from 0.5 hours to 1.5 hours with a median duration of 50 minutes. The interviews were recorded and transcribed. The distribution of informants is shown in Table 1:

The first and the senior authors’ long-term experience of working with managing and improving cancer care in university hospitals was a source of knowledge to the field studied and influenced how we prepared and conducted interviews, and how we interpreted the data. In line with Berwick [52], we consider this an advantage. Reflections on this aspect of the study were documented in a separate essay during the research process.

The process of identifying core characteristics of cancer diagnoses, patient groups and type of hospital that have an impact on the construction and execution of CPPs included analyzing transcribed interviews and written sources, reflecting upon our own experiences studying literature on ICPs, CPPs and coordinated hospital care in general. All together, the goal was to develop analytical models that structure our data to address our research question. Gradually, we developed the three approaches presented in the introduction. By then we had started to structure interview data through exploratory coding using NVivo (QSR International, Melbourne, Australia). As our analytical models emerged, we supplemented the NVivo analysis with new nodes and new layers of nodes. Qualitative and quantitative data from written sources were compiled in tables structured by variables thought to be relevant for the analytical dimensions of the analytical approaches. The synthesized categories presented in the tables in the results section are thus based on data from several of our available sources.

## 3. Results

**Figure 1:**
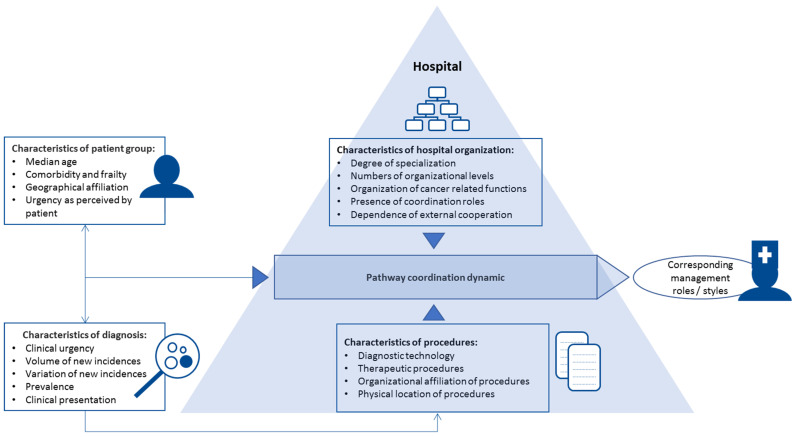
Variable groups with indicators.

The presentation of results is organized according to the two independent variables: hospital and diagnosis. For both, we have data on several core variables representing the dimensions in our analytical approaches. The core variables are directly and indirectly indicators of complexity and variation in predictability. The main variable groups and their connection with each other are described in Figure 1.

### 3.1. Hospital-Related Variations

We start by presenting general relevant information about the four hospitals and then show data from the three pathways attached to each of the hospitals, followed by data of patient groups in each of the pathways and finally data based on properties of each of the three diagnoses. Each of these table-based approaches is commented, summarized and supplemented with citations from the interviews. This provides us with an overview of the premises for identifying variations in coordinating conditions in and between pathways for different diagnoses and types of hospitals and thus establishes a platform for discussion of managerial and organizational consequences. We start in Table 2 by presenting relevant data describing cancer care at the four hospitals included in this study.

There are considerable differences in the number of patients diagnosed and starting a standardized CPP among the four hospitals but considerable number of cancer patients are treated even at the community hospitals. The difference between the two university hospitals and the two university hospitals in number of patients treated surpasses, however, the differences expressed in the table since the university hospitals also receive patients for tertiary care. Nevertheless, the capacity of the community hospitals to deliver coordinated and appropriate cancer care is indicated by this citation:

“Community hospital D is of the right size, there are short communication routes, there is the right number of specialties in the hospital, but still it’s easy to reach out to. It is not so big that you’ll lose track here. However, a hospital shouldn’t be too small because then there will be too few specialties and too few with cutting-edge competence.”

(D4)

The university hospitals have a combined role as both specialized regional care providers and community hospitals for surrounding districts. For several reasons, the process complexity increases in general as we go from community hospital to university hospital. The number of units involved increases, as does the degree of sub-specialization and the presence of formal and informal subunits, especially in the diagnostic units and the oncology departments. This experience is expressed in the following citation:

“I believe it’s simply that the silos are becoming bigger. When you’ve got more hospitals, each hospital can be seen as a silo. Then you’ve got smaller silos within the hospitals. So I believe it’s as simple as the organization is more complex, and that you have more of those lines or silos to deal with.”

(A24)

The increase in multimodal treatments and referrals, and the subsequent traveling of patients between hospitals, increases complexity. Complexity can also decrease when going from a community hospital to university hospital. The following citations refer to one of the university hospitals having two sites, thus sheltering cancer activity from emergency care.

“Specialization in the diagnostic functions and also the fact that we are allowed to be a distinctive elective hospital specialized in cancer care is extremely important. To run a business like this in addition to, for example, emergency operations would reduce the quality of care in my eyes. So we are, as we see it, extremely lucky to have the position we have.”

(A22)

“That hybrid model is very difficult to handle. The acute care pathways destroys the whole planned structure that a top-notch competence need. Unpredictable, have to constantly run around. All the plans you’ve made, you must make again because they didn’t work. And we live with these challenges on a daily basis. And if you then collaborate closely with other areas that are more electively run and that have structure and order that also have local hospital patients, who will take care of them?”

(C7)

“I saw the operational benefits of a sheltered elective arrangement. When you received referrals or attended an MDT meeting and planned four, six, eight weeks ahead in time, versus our internal arrangement where you experience these fluctuations that are not balanced to the acute flow of patients, but at least there was not the large amount of benign surgery where the waiting lists are 18 months for many patients no one want to operate. It’s surgery that means an intervention in their lives and should be planned well in advance. When we try long-term planning, the CPPs come and mess this up.”

(A4)

Only university hospital A has a specific comprehensive cancer coordinating entity. The patient coordinating positions that are mandatory for the standardized CPPs in Norway are organized at the central hospital level at the two community hospitals. In these hospitals, these navigators coordinate the steps of the entire pathway in their hospital regardless of which unit is performing the task. In contrast, the two university hospitals have separate navigators in each clinical department involved in the pathway and they are organized in each unit. The dynamic of pathway coordination between levels of hospitals was described like this:

“If there is someone you need to discuss or create an individual path for, you could just call and discuss and make an agreement, and that’s also how it works with the referring hospital, that is, if there’s anything they want, they’ll call. It does happen that one is unsure about something, that they’ll call from the community hospital and explain why they absolutely want to do it in that way.”

(A4)

However, sometimes this system of improvising networks may have some limits in reaching solutions:

“In our hospital there are three persons who work with colorectal cancer as their primary task, and they know the environment at different locations in university hospital A, but in the management line there is not so much contact and I think that when we have a bottleneck, we would maybe benefit from having some arenas where the leaders could meet. The leader arenas that exist are clearly tied to level one or level two. There are not many meeting points at level three or four across the hospitals.”

(B1)

To further investigate the hospital-related variation, we looked into the hospitals’ activity connected to each of the three diagnoses under study, beginning with the breast cancer pathway, illustrated by monitored activity of the standardized CPPs in Table 3.

The number of patients in the breast cancer pathway differs substantially between hospitals A and D. In this pathway, diagnosis and surgery are defined as community hospital tasks. Radiotherapy is centralized. The patients are recruited from two channels: the national screening program or investigation prompted by a clinical finding. Both groups are referred to a breast diagnostic center at each hospital dedicated for this purpose only. If the patient is diagnosed with cancer, the majority will start treatment at their community hospital, and receive radiotherapy if needed at a university hospital. The exception is patients with (locally advanced, stage III) tumors. These patients are referred to the university hospital and undergo an MRI of the breast before starting adjuvant chemotherapy. All four hospitals have MDT meetings, although oncologists do not participate at university hospital C. In all four hospitals, a breast surgeon has a coordinating role on all issues related to CPP governance. However, only in community hospital D are the units involved in breast cancer pathways located at the same site.

The differences in the volume of patients diagnosed with colorectal cancer (CRC) do not vary to the same degree as for breast cancer, as shown in Table 4.

For patients with localized disease, the colon cancer pathway is managed at the community hospital. Corresponding departments manage diagnostics and treatment in all four hospitals. The MDT meetings consist of radiologist, pathologist, gastro-intestinal surgeon and oncologist. Gastroenterologists are only present at community hospital B as they are the ones conducting the colonoscopy. If the cancer has metastasized, or in the case of locally advanced rectal cancer, the diagnostic and treatment procedures are performed at a university hospital if considered curable. Colorectal cancer patients with metastases, usually in the liver or lung, are discussed at MDT meetings at the university hospital comprising members according to the specialties involved. Patients with operable metastatic disease have surgery at the university hospital. The pathway in a community hospital of a CRC patient was described like this:

“With metastatic colorectal cancer some of the patients are to have neoadjuvant chemotherapy so then there’s a consultation and the patients’ information is sent and the patients are discussed at an MDT meeting in university hospital A and then a path is planned, for example, if they are to have both rectal surgery and liver surgery and that they’ll get neoadjuvant treatment with us, and then a time path is created and in many ways I think that works very well.”

(B1)

Community hospital B is the only hospital where all functions related to this pathway are gathered in one location. At university hospital C and community hospital D, colonoscopies are performed in several locations. At both university hospitals, the gastro-intestinal surgeon has a coordinating role concerning medical related topics and in overall pathway governance.

The variation between the four hospitals concerning ovarian cancer is shown in Table 5.

The diagnostic procedures in cases of suspected ovarian cancer shall, according to the standardized CPP, be made by a gynecologist at a specialized department of gynecological oncology at a university hospital. The proportion of patients diagnosed with this cancer at university hospital A is high. The initial management of these patients is delegated to the gynecology departments at the community hospitals. Occasionally this is done post-surgery after an abdominal intervention at a community hospital. The university hospitals are responsible for the majority of cases, including detailed diagnostics work-up and treatment—both surgery and chemotherapy. In addition to gynecological oncologists, pathologists and radiologists attend the MDT meetings. The head of the gynecological oncology departments also acts as a coordinating officer for all medical-related purposes. All involved specialists at the two university hospitals are co-located at the hospital areas. The following citation describes how this works in practice:

“We have regional meetings, so there is an oncologist in addition to a radiologist and a doctor from nuclear medicine and pathologist. And it depends what else we need. That is, if we need anesthesia or a gastro-intestinal surgeon or a sarcoma surgeon or any need in particular.”

(C9)

### 3.2. Diagnose Related Variation

In Table 6, which is organized according to diagnosis, we have extracted data representing patient-related variables that may influence the preconditions for achieving coordination and thus the implementation of CPP processes. These variables are volume of patients, medical urgency expressed by stage and relative survival and risk of comorbidity expressed by median age of patient population.

**Table 6:** National data characterizing the patient groups of the three diagnoses studied [57].

		Breast Cancer [58]	Colorectal Cancer [59]		Ovarian Cancer [60]
			Colon	Rectum	
			Female, Male	Female, Male	
Incidence	Total	3753	2979	1316	528
2019			1541, 1438	539, 777	
Screening		Women 50–69 years old [58]	2019: no screening [59]		High risk-groups [60]
Fraction of patients by stage * 2015–2019	I	42.7%	17.9%, 18.9%	25.1%, 24.9%	20.3%
	II	32.9%			
	III	10.6%	52.1%, 51.3%	44.7%, 46.2%	20.7%
	IV	4.1%	22.3%, 23.3%	19.3%, 19.4%	52.0%
	Unknown	9.8%	7.7%, 6.5%	10.8%, 9.5%	7.0%
				
Median age at diagnosis 2015–2019		62.0	73.0	70.0	67.0
5-year relative survival by stage * 2015–2019	Total	92.0%	71.1%, 68.1%	71.5%, 71.1%	50.3%
	I	100.9%	98.9%, 98.3%	96.1%, 98.0%	97.4%
	II	96.1%			
	III	79.4%	85.4%, 84.4%	80.8%, 82.3%	61.9%
	IV	34.0%	20.8%, 15.1%	24.0%, 20.4%	37.1%
	Unknown	78.2%	35.3%, 31.4%	46.0%, 46.3%	40.5%
				

* Stage indicates how advanced the cancer is.

When comparing the three diagnoses, we recognize that breast cancer has the highest volumes, the highest frequency of patients diagnosed in stage I and the lowest in stage IV (metastatic disease), the highest expected relative overall survival for patients in all stages, and the youngest patient group. In contrast, compared to breast cancer, ovarian cancer patients are fewer (one-seventh), the cancer is diagnosed in more advanced stages, and survival rates are worse for all stages. CRC patients are the oldest population and comorbidity is expected to be higher in this population. For the CRC population, 15–25% of the patients presented with acute abdominal symptoms. As we see in Table 6, there is variation in the stage of the cancer at presentation and we find more advanced disease (stage III and IV) in ovarian cancer and CRC patients compared to breast cancer patients. Nevertheless, patients’ subjective experience of urgency may be higher for a possible breast cancer patient, as one physician explained:

“Because I worked for a long time, I started with breast cancer and had both colorectal and breast cancer patients, and we had to get these breast cancer patients in before the colorectal cancer patients, because I believe it has to do with this is something you feel, it’s outside the body, and the breast cancer patients were more impatient than female colorectal patients who were more relaxed in a way.”

(A2)

The characterizations and presentations are further elaborated in Table 7, together with other relevant information that may add to the complexity and predictability of the CPP.

**Table 7:** Clinical presentation, diagnostic work-up and treatment.

	Breast Cancer [58]	Colorectal Cancer [59]		Ovarian Cancer [60]
		Colon	Rectum	
**Clinical presentation** Characteristics and presentation of signs and symptoms	Visible or palpable lumps or changes in skin or tissue Changes seen on mammography screening	Ambiguous symptoms Tumor/polyp on ano-/rectoscopy/colonoscopy Acute intestinal perforation, bleeding or ileus		Ambiguous symptoms Acute ileus or thrombo-embolic event
**Diagnostic workup** Essential procedures and technology	*Triple diagnostics:* Clinical examination Clinical mammography and/or ultrasound (radiologist) Fine-needle aspiration cytology (FNAC)/cyst puncture/core needle biopsy (CNB)/vacuum-assisted core biopsy (radiologist, examined by pathologist) *If incomplete needle biopsy or malignant finding:* Open biopsy (surgeon) *Neoadjuvant treatment:* MRI breast (radiologist)	Clinical examination with digital rectal exploration (DRE)/rectoscopy Colonoscopy with biopsy (gastroenterologist, examined by pathologist) *If incomplete* *colonoscopy:* CT-colography (supported by radiologist) For TNM * stage: CT thorax, abdomen and pelvis (radiologist) *Acute presentation:* CT abdomen and pelvis (radiologist)	Ano-rectoscopy with biopsy For TN * stage: High-res MRI with surface coils (radiologist) For T1/T2 * stage: Ultrasound rectum (surgeon) For M * status: CT thorax, abdomen and pelvis (radiologist)	CT thorax, abdomen and pelvis (radiologist) Blood sample Genetic test Clinical and gynecological examination with ultrasound (UL) (gynecologist) Calculation of Risk of Malignancy Index (gynecologist) *Stage III-IV:* UL-guided biopsy(gynecologist and radiologist, examined by pathologist)
**Treatment** Essential procedures	Conventional breast conservation surgery (BCT)/oncoplastic breast conservation surgery (OBCS)/ablation/mastectomy/sentinel lymph node biopsy (SNB)/axillary dissection (AD) Radiotherapy *Adjuvant and neoadjuvant systemic therapy:* Hormone therapy/ Chemotherapy/ Targeted medical therapy	Lymph node dissection/colon resection/dissection in circumference of tumor Adjuvant chemotherapy *Acute presentation:* Resection with or without anastomosis/ colostomy/stent	Total mesorectal excision (TME)/Partial mesorectal excision (PME) *Neoadjuvant and adjvuvant therapy:* Concomitant radiotherapy and chemotherapy	Cytoreductive surgery/fertility-preserving surgery Neoadjuvant and adjuvant chemotherapy
		*Palliative treatment:* Palliative surgery Palliative radiotherapy		

* Tumor (T), Node (N), Metastases (M).

Table 7 demonstrates that patient referral is referred to the hospital involves two dimensions. One dimension concerns whether hospital referral results from a screening program or from an incidental or symptom presentation to the general practitioner (GP) or an unexpected finding during an unrelated surgery. In 2019, breast cancer was the only diagnosis among the three diagnoses studied, with a national screening program. The other dimension is whether the admission to the hospital is acute or planned. An emergency tag to mobilize resources is also in use in elective cancer patient pathways. The clinicians label the radiology or pathology form with “citu” to have it prioritized. In addition, the perceived state of urgency may also be influenced by the stage or aggressiveness of the cancer. The variations among patients within a diagnostic group make the preconditions for standardized processes more complicated.

Table 7 also shows that the diagnostic work-up procedures vary substantially between the three diagnoses. For breast cancer patients, the diagnostic work-up may be completed with a mammogram and an ultrasound-guided fine needle biopsy, plus an MRI for patients with locally advanced tumors. A patient with suspected CRC will need a colonoscopy and a complete thoraco/abdominal CT scan before surgery. Patients with suspected ovarian cancer will also undergo a complete thoraco/abdominal CT scan, but final diagnosis is based on the operation specimen.

Variations in surgical procedures within and between these three diagnoses appear as well from Table 7. Tumor resection is straightforward for breast cancer in the majority of cases but will often need concurrent or secondary reconstructive procedures. Locally advanced rectal or ovarian carcinomas may require extensive tumor resection and a broader competence in the surgical team while CRC might require highly specialized teams for metastasectomies either in lung or liver.

As multiple factors related to the diagnoses influence the diagnostic and treatment procedures performed, the organization of the pathways is also affected. The characteristics of organization are depicted in Table 8.

Table 8 shows that independent of hospital, there are important variations in the organization of the main steps in the pathways of the three diagnoses studied. These variations influence the context for coordination by creating complexity and the type of work process performed and the kind of competence in charge at the various steps of the pathway. For the initial part of pathway when the patients are admitted to the hospital, the organizational pictures are: Breast cancer patients are all received at a breast diagnostic center where all resources and competences connected to diagnostic procedures are gathered. These centers are also sheltered from activity related to other patient groups. At all four hospitals there is no easy access to MRI technology. For patients with suspected CRC, there is no designated diagnostic center. The colonoscopy facilities are also used for other patient groups. However, there may be a number of slots every week reserved for patients with suspected CRC. In university hospital C and community hospital D colonoscopy are performed also outside the main hospital site. Patients with suspicion of ovarian cancer typically arrive at a department of general gynecology. An interviewee described the interaction between university hospital A and community hospitals related to the ovarian pathway:

“It’s up to our department head to contact the head of the local gynecological department when something is not working, to put pressure on the person in question so that things go faster. But I have to say, it’s noticeable that for these departments, cancer is only a part of their task. They have a lot of births and do a lot of other things as well. So it’s not always the case that cancer is perceived to have the highest priority.”

(A8)

Several of our informants expressed a wish for developing towards organizational structuring supporting an integration of cancer pathway related specialties, expressed like this:

“If we were more like a cancer hospital where we could have gastro surgeons, gastro oncologists and palliative professionals more integrated, so that the patients could stay with us like they do in other diagnosis groups like breast cancer, lymphoma, and sarcoma, they belong to the cancer department their whole pathway.”

(C16)

The task split between community and university hospitals varies between the diagnoses. The majority of breast cancer patients are offered surgical treatment at their local hospital—either a community or a university hospital. Only the patients with locally advanced disease are referred to a university hospital, and preferably neoadjuvant chemotherapy, before surgery. The majority of CRC patients’ entire pathway is at the local hospital. However, patients with locally advanced rectal cancer or metastasis deemed resectable are referred to the university hospital. Treatment for locally advanced ovarian cancer patients is centralized at specialized gynecological oncology departments at the university hospitals. The cooperation along pathways across hospital borders is in addition to be influenced by the functional division of labor in clinical treatment significantly influenced by differences in roles and competences in the diagnostic support disciplines of radiology and pathology. Two informants express it like this:

“My perception is that many specialties are quite clear, such as what they say in community hospital D: “this is what we are doing here, and we are sending these patients away to the level above.” However, in radiology and laboratory, it has become the case that one has to be able to serve the whole spectrum—everything in diagnostics and controls regarding these patients. Even if the patient had been referred to a higher level of care, there is little to say about what we are doing. In a way, we have to follow them the whole pathway.”

(D2B)

“Because our radiologists and pathologists are dedicated to one field, whereas if you work as a radiologist or pathologist in a community hospital, you need to know all sorts of stuff, which doesn’t make it strange that one can disagree and assess things differently.”

(A8)

## 4. Discussion

We assume that specific features of patients, hospitals and diagnoses influence the contextual framework for achieving coordination of CPPs in hospitals. Since these features vary depending on diagnosis and hospital type, understanding the characteristics of these variations is of value for management of CPP coordination.

### 4.1. Horizontal Coordination—Differences in Complexity and Predictability

In line with a project management approach [33] to context and coordination, we base our analytical approach on the assumption that higher complexity combined with more or less predictable variations make coordination through standardized CPP more difficult to implement [32]. The combination of complexity and unpredictability requires room for improvisation and flexibility not to reduce adherence to quality standards for the pathway process on neither single patient nor the institutional level. Across our four hospitals and three diagnoses, we identified four elements that affected complexity and uncertainty. They typically relate to different sources of uncertainty [31], characteristics of patients, process and organizational context.

First, there is an unpredictable variation in patient volume. According to the literature on standardizing processes, greater stability [3,18] and volume [19,21] support the conditions necessary to implement standardized pathways. In the current work, breast cancer has the highest volume of the studied diagnoses, as well as the most stable inflow of patients over time due to the national screening program, which recruits more than 50% of total breast cancer patients. Colorectal cancer is also a cancer type with high patient volumes. However, there is higher variation in referrals to hospitals and in referred suspected cases of CRC that actually end up with a cancer. Ovarian cancer is a less common cancer with relatively lower variation in referrals over time.

The second contextual variation affecting coordination is control versus competition for core resources. This is influenced by hospital organization and whether the resources are sheltered from other priority tasks, especially emergency activities. This variation concerns the relative fluctuations of supply and demand, of relevant resources for the diagnostics and treatments needed in each pathway, the degree of urgency normally present for patients with each cancer diagnosis, and the organization of hospitals. This means how ownership of the units controlling limited resources is organized in relation to the units in need of them. Organizational dimensions decide whether resources related to a cancer pathway are sheltered or have to compete with other diagnoses and pathways. Breast cancer is diagnosed and treated at dedicated breast diagnostic centers at all four hospitals. The surgeons that perform cancer surgery are not involved in emergency activities. The specialized rectum cancer surgery teams and metastases surgery team at university hospital A are sheltered from acute gastrointestinal surgery activity. The specialized gynecological cancer departments at both university hospitals are sheltered from the general gynecological activity. In university hospital A, this department is located at a specialized cancer hospital. In contrast, colon cancer surgery at all four hospitals is integrated and organized with the other GI surgical activity, including a high degree of acute care activity. Medical urgency increases the coordination challenge of mismatch between demand and supply of resources for a cancer pathway. Ovarian cancers are more commonly diagnosed in more advanced stages than breast cancers and colorectal cancers and thus have a higher degree of medical urgency when it comes to receiving necessary resources in a timely manner. The medical urgency of ovarian cancer is also reflected in the lower expected relative survival rates. However, as we have illustrated, patients’ experienced urgency may not parallel with medical urgency.

The third contextual feature affecting complexity and predictability is connected to the clinical presentation, the diagnostic workup, and the therapeutic procedures in each specific cancer diagnosis. The general complexity increases when regional/locally advanced (stage III) or metastatic disease (stage IV) is detected. Stage III and IV patients (not colon for stage III) are admitted to the university hospitals for further diagnostics if radical surgery is deemed possible. The frequency of locally advanced and metastatic cancers is higher in ovarian cancers than in the two others, and the majority of the patients are treated at the university hospital. The diagnostic work-up of colorectal and ovarian cancers includes more diagnostic and specialized imaging procedures before surgery, which adds to the complexity of these CPPs. All three cancers may have ambiguous symptoms. However, the majority of diagnostic processes for breast and colorectal cancer seem to be straightforward in most of the cases. The breast cancer pathway is the most standardized in accomplishing the primary diagnostic workup. However, this pathway is also more developed when it comes to introducing alternative treatments based on precision medicine, which then depend on a more precise and complex radiology or molecular pathology analysis.

The fourth contextual dimension influencing coordination capabilities is variation in comorbidity and frailty. The patient’s total disease burden may increase complexity and the need for individualized treatment for patients. Comorbidity is related to age and colorectal cancer patients have a higher median age than the two other cancer types.

The fifth element originate from the organizational context of CPP. On one end, in breast cancer pathway the vast majority of patients are treated at their community hospital. While on the other hand there is the ovarian cancer where the majority of patients have pathways including two hospitals in the same way as for locally advanced/curable metastatic CRC.

To summarize, our findings show that the five groups of variables that influence horizontal coordination in CPPs are differently weighted in hospitals and diagnoses. Comparing type of hospitals, this seems to be the tendency: Streamlining might be easier in university hospitals due to higher patient volumes. However, these hospitals have more complicated organizational structures, a broader case mix and higher proportions of more advanced cancers, all of which increase complexity and thus influence the conditions for coordination. The challenges of coordinating CPPs in the university hospitals seem to decrease when cancer procedures are sheltered from other activities, especially emergency activity.

**Figure 2:**
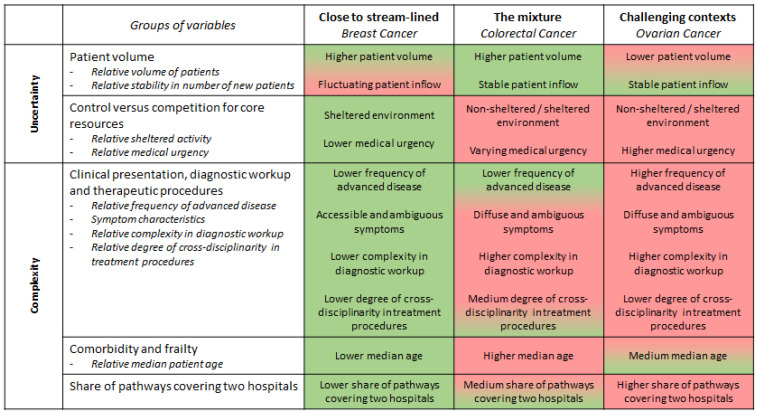
Three diagnoses expressing two positions and one mixture to: what makes the difference? (The degree of unpredictability and complexity is expressed using green (lower) and red (more extensive) colors.).

The main trajectories of differences between diagnoses and their consequences for challenges in accomplishing coordination and standardization are outlined in the Figure 2.

In this figure the first two group of variables affect predictability of the CPPs and the three next groups of variables are influence the complexity related to CPP performance. From this, we derive that breast cancer CPP of the three will be most suitable for described and practiced closest to a standardized programed chain of events and procedures. We do not have any information about the prevalence of organized CPP in different cancer diagnoses. Nevertheless, in a review [5] of studies on integrated care planed implemented in cancer care breast cancer by far is the diagnosis most frequently selected as the subject of a study.

### 4.2. The Dynamic of Processes—Does One Pattern Fit All Patients, Pathways and Hospitals

As outlined in the introduction, ICPs and CPPs emerged from an interpretation of pathways as linear sets of procedures that can be described using flowcharts [1,10,11,28]. Our findings do not disprove that elements of these programmed chains are present for all three pathways. The national documentation of rough verbal descriptions of standardized pathways, as well as the standardized flow chart descriptions developed and published in the quality system of university hospital A, are constructed to show the programmed chain of action and decisions. However, there is neither a technologically supported work or information flow, nor an organizational formal structure aligned with the prescribed programmed chain. The documented standardized CPPs therefore play a role as a kind of soft governance infrastructure facilitating reconciliation of the involved actors’ interpretations of the steps in the chain of actions. The CPP is not a blueprint that everyone is obliged to follow but a reference according to which the involved partners communicate and negotiate. Thus, it supports coordination along a chain of events that still needs active interventions to succeed. This way of understanding CPPs also dissolves the apparent contradiction between the claim, on one hand, that they reduce complexity and unpredictability and the claim, on the other hand, that they do not reduce these issues but rather make it possible to adapt to them. By establishing a common language, CPPs reduce the complexity of communication around a still complex process. This common reference of a language then facilitate cooperating behavior necessary to cope with a process of complexity and unpredictability. Since the hospitals’ basic organization and management lines are not aligned with the process chain of CPP procedures, the deployment of the soft governance expressed by a standardized CPP must be performed by a management function linking the elements together through a stewardship [50].

In addition to affirming the presence of real programmed chains of action, our study shows that other types of coordinating dynamics are present. We connect them to the concepts of consultative hub and problem-solving web [41]. Some procedures, both diagnostic and treatment-related, are complicated and iterative with cross-disciplinary involvement. Examples of such cooperation include the examination of combined diffuse symptoms of patients with suspicion of one cancer diagnosis; the involvement of specialists in gastrointestinal surgery and internal medicine when discovering suspected cancer during a colonoscopy; the cooperation of breast surgeons and plastic surgeons during primary reconstructive breast surgery; and the joint efforts of gastro-surgeons with urologists, plastic surgeons or even orthopedics in cases of locally advanced rectum cancer. An interesting special case of consultative web is the work of gynecologic oncologists, who exemplify the unification of multi-disciplinarity in one highly specialized medical doctor (MD). They are skilled in both gynecological surgery and medical oncology; they take biopsies and also perform abdominal surgery. Thus, while they are masters of multiple trades attached to the fields covered by their hub, they also act as a consultative hub, drawing on other specialists.

What kind of specialist inhabits the core roles of the hub- and web-processes is not indifferent. Does she have a background providing her with a general cancer competence? Our findings indicate that this, largely, depends on who runs the technology used during examination or treatment. Radiologists play a dominant role in initiating the breast cancer CPP. They are in charge of the mammography and ultrasound technology and run the breast diagnostic centers. Consultants in gastrointestinal medicine are in charge of the colonoscopy technology and are therefore the key players in the initial phase of the CRC pathway. Gynecologists manage the admission of lots of the ovarian cancer CPPs, either as general gynecologists or specialist gynecologic oncologists. They either perform or organize both the examination and treatment procedures. Post-treatment, the patients are generally followed by the specialist responsible for the first treatment. Thus, a patient who receives surgery first has their follow-up managed by the surgical specialist, while a patient who receives neoadjuvant treatment first has their follow-up managed by an oncologist. We suggest, however, that the various specialists do not necessarily have the same in-depth and broad knowledge of the specific disease and the interplay of treatments and thus are unable to coordinate the medical process, the logistical process and the patients’ need for comprehensive communication

The MDT meetings, too, are examples of consultative hubs in practice, albeit to varying degrees. Morris et al. [12] argue that teaming processes are crucial to the successful outcome of a pathway program in gynecological cancer. Elements of consultative hubs are present not only in certain sequences of the pathways, but also as iterative processes during the pathway. This is in line with explanations by May et al. [54] and Shiell et al. [27] of how complex interventions in health care actually work. This finding also corresponds to the arguments made by Trosman et al. [1] in their study of coordinating complex task interdependence in cancer care. As Slack et al. [32] states, high complexity challenges the ability to deploy traditional governance control in project-like tasks, and Plowman et al. [48] then argue that control, as a management mechanism, should be substituted by enabling. This is not least what we then expect to find in CPPs with existing solution hub elements.

The third type of coordinating dynamic we introduced was problem-solving webs. In line with other scholars studying healthcare [39,41], we found that connecting provided a crucial supplemental dynamic in the pathway. These networking processes consisted of both the relational work itself and the bargaining efforts this work entails. This dynamic is about connecting capacity to needs for resources, connecting information requirement to access to information, connecting professional knowledge to knowledge gaps and connecting what makes sense from one logic to another. These connecting activities are performed partly according to a system, partly based on routines and partly from artistic improvisation; they are all based on network processes. Network activities [39,41] can both connect and align context elements to needs in a programmed chain and may connect several elements with different coordination dynamics in the same pathway. An example of the latter is connecting steps in a linear sequence of events and procedures. This may as well connect logistics and outcome from different solution hubs along a pathway or even iterative elements of diagnostic and treatment procedures, effect examinations and cross-disciplinary counseling. The problem-solving webs is close to its pure form in the pathway coordinator office at the two community hospitals. The employees in these units have their major legitimacy and work related to connecting information and demanded resources to patient and to the physicians present at any time in the CPPs [25]. Handling variation in predictability combined with complexity challenge the ability practicing pre-planned processes [32] and require management characterized by brokering [50] related to information and access to resources and to building communication channels suitable for this performance.

**Figure 3:**
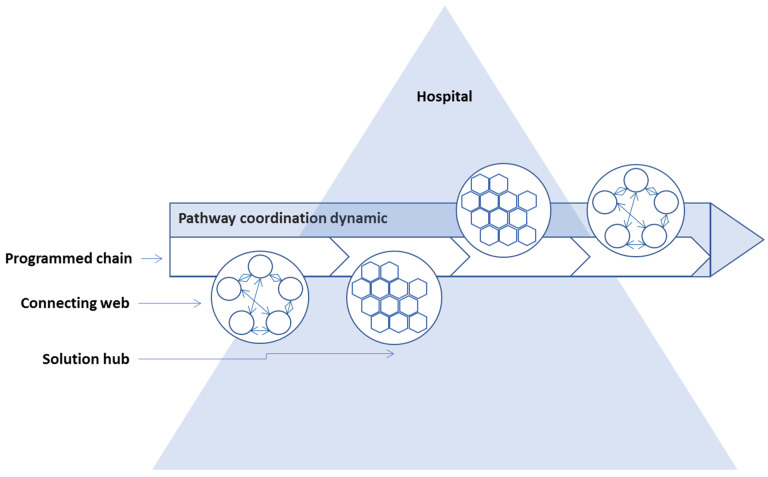
Combination of pathway dynamics in horizontal coordination processes of CPPs.

Finally, in the discussion of different types of process dynamic, we return to Figure 2 delivering a summary of the different conditions for performing horizontal coordination when comparing the three diagnoses. From this picture representing different profiles of complexity and unpredictability we may derive the following: Though elements of all three process types may be present in all three diagnosis-based CPPs as illustrated in Figure 3, processes of solution hubs and connecting webs and the associated management requires are more prevalent in ovarian CPP and partly in CRC CPPs compared to breast cancer CPPs.

### 4.3. Characteristics of Hierarchical Coordination in and between Hospitals

Those actors representing each step during a pathway may coordinate their activity by combining several well-known measures such as internalized competences, imposed rules, standards and guidelines and through mutual negotiations, adjustments and improvisation. However, these measures are not always enough to reach a solution, leading to the need for the involvement of higher hierarchical levels representing the economic–administrative logic. For pathway governance, a key question is whether there is a medical stewardship [61] representing the professional community and the medical logic that encompasses the entire CPP and can communicate in a balanced way to representatives of the economic–administrative logic. To some extent, we identified professionals with a comprehensive responsibility for the pathway of each cancer diagnosis, usually a senior physician. In university hospital A, there is a coordinating cancer center board as well as a pathway coordinating stewardship for each CCP that reports to the board and has a mandate to facilitate coordination and solve bottlenecks. However, the extensive lack or weakness of semi-formalized stewardship roles covering the CPP across borders of the formal organizational entities challenge the ability to create a clear meeting points creating opportunities for mutually explorative and negotiating processes between the professional medical logic and the economic administrative logic.

**Figure 4:**
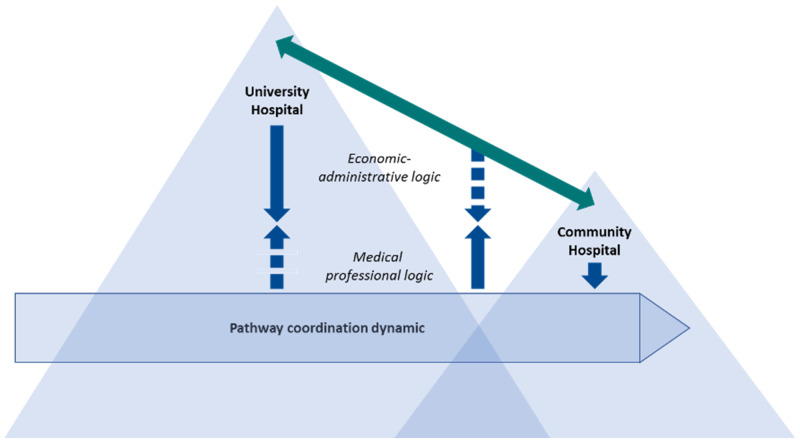
Vertical coordination in processes in and between hospitals. (The green arrow expresses the institutionalized interaction between top management in the hospitals. The blue arrows indicate vertical interaction.).

A second level of coordination challenge exists between hospitals. In line with the general analyses of Axelsson and Axelsson [62], this is a prevalent phenomenon in pathways moving between community hospitals and university hospitals. Although there is a lack of actors with coordinating authority representing the comprehensive medical professional community speaking up on behalf of the pathway, there are many informal and semiformal networks between medical communities across hospitals [25]. However, in the current case, there is a lack of coordinating agents representing the economic–administrative systems of the cooperating hospitals. The top levels of the hierarchical management in each health region meet, but they lack the capacity to manage and support the needs of coordination activities on what we might call the medium level of the hierarchy. The coordinating interactions happening vertically through the hierarchical levels in and between hospitals are shown in Figure 4. The dotted lines illustrate a lack of functional vertical coordination. Thus, we illustrate limited integrating management between institutional logics on the borders between hospitals cooperating along the same CPP. To a greater extent this creates a challenge in the CPPs where relatively more of the patients’ pathways cover two hospitals; thus, being more prevalent in ovarian cancer and parts of the CRC CPPs than for breast cancer CPPs.

## 5. Conclusions

In the current work, we have systematically described how contextual variables affect the premises of CPP coordination. We argue that both the diagnosis and the type of hospital make a difference. We have also identified different variables that create difference in coordinating premises. Using three analytical approaches allowed us to better understand the mechanisms contributing to variation in coordination practices in CPPs. From this, there are two lessons learned.

First, it is necessary to recognize that CPPs are characterized to varying degrees by a combination of contextual and procedural complexity and variation in predictability. This will influence the premises for executing the necessary horizontal coordination along the pathways for single patients and of the flow of patient groups within and between hospitals. There is a need to acknowledge that CPP coordination involves more than linear sequences of simple events. To varying degrees, it is also characterized by the dynamics of consultative hubs and problem-solving webs, which, fundamentally challenges the basic assumption that ICPs are standardized processes in an industrial sense. The balance and combination of dynamics from these three categories of processes varies across and within cancer care pathways. This variation should be mirrored in the way CPPs are documented, organized and led. Performing management has to adapt to these variations if CPP coordination is to be successful. One size does not fit all pathways, their constituents or types of hospital. CPPs should be organized and led according to an understanding of the specific diagnosis, type of hospital, and patients being treated. Thus, all hospitals need management that engages in controlling, enabling and brokering, in addition to having a general integrating role. To avoid the challenges that mixed management styles generate, hospitals may develop specialized and sheltered units. This has been done to some extent at university hospital A. However, for the many patients with pathways crossing the borders of the coordination typologies this could be an unsatisfying solution. In line with a conclusion of Cook et al. [41], we also anticipate that limited overall integration will impair the ability to promote a learning environment.

Second, the need for vertical coordination of processes in and between hospitals related to CPP implementation address the need for specific managing roles and skills. Professional–medical and the economic–administrative institutional logics meet when topics from pathway coordination are raised to a higher organizational level. Collaboration and negotiation between the logics depends on the existence of connecting points and on the logics having representatives with legitimate authority being present at these points. If such representatives do not have a formal role, a steward or an ambassador should be appointed. We call for the acknowledgement of the need for such a stewardship role representing the professional–medical logic in these pathways in connection with the hierarchical line management in the hospitals. In parallel, we recommend that an integrating ambassador represent the management line where connecting networks of pathways cross hospital borders.

In line with Zuiderent-Jarek [37], these two arguments together contribute to an understanding that CCP coordination measures should be developed based on a situated platform. Within this frame-work we suggest future implementation and improvement of CCPs and ICPs to consider what is apt to be standardized and what should be kept flexible and be influenced by the type of characteristics of the specific diagnose, patient group and context. Secondly, the improvement of CCPs should pay more attention to development of suitable formal and semi-formal structures to connect the mixture of hubs, webs and chains present along the pathway in addition to promote cooperation between hierarchical levels and institutional borders. Finally, the improvement of CCPs and ICPs should focus on which type of professional cancer related background and management style is required to fill the key coordinating roles in different parts of the pathway. The necessity of accomplishing these improvements may be reinforced by the increasingly coherent process of providing more precise diagnostics, by the need for advanced information to identify targeted therapies, and by the more in depth follow-up to measure effects of treatment.

Finally, further research on ICPs in general, and on CPPs specifically, evaluating their effects and how they work should consider the interplay between structural contexts, the features of what is being coordinated through CPP, as well as outcomes. We argue that the direction of CPP research will be more valuable for developing the concept and improving CPP implementation than continuing to view CPPs as a single standard intervention and measuring outcomes before and after or comparing cases of implemented CPP with cases where a CPP was not put to use. Knowledge on complex interventions involving complex tasks in a complex system with complex sets of outcomes introduced in a variety of contexts of health care systems should be built using other types of research approaches and applying other types of research methods.

## Figures and Tables

**Table 1:** Number of informants from the participating diagnoses and hospitals.

	Hospital 1	Hospital 2	Hospital 3	Hospital 4	Total
Ovarian cancer	5	4	2	1	12
Breast cancer	6	4	2	1	13
Colorectal cancer	10	9	4	3	26
Two or three cancers	3	1	4	7	15
Total	24	18	12	12	66

**Table 2:** Data characterizing cancer care at the hospitals included in the study.

General Characteristics	University Hospital A	Community Hospital B	University Hospital C	Community Hospital D
No of patients newly diagnosed with all cancers	4715	1340	2545	513
Population served	Local: 550,000 Regional: 3,000,000	Local: 250,000	Local: 460,000 Regional: 1,100,000	Local: 110,000
No of patients starting standardized CPPs	5985	3134	4235	1005
No of organizational levels involved	4–5 levels	4 levels	3 levels	3 levels
No of units involved	Level 2: 12 of 15 units Level 3: 31 units	Level 2: 3 of 7 units	Level 2: 11 of 22 units	Level 2: 2 units Level 3: 7 units
No of cancer care sites	4 sites	1 site	3 sites	3 sites (2 satellites)
Coordinating function of cancer care	Cancer Center Board organized as a leadership matrix	Head of one division has a major coordinating role	The hospital management and medical director	Medical director coordinating role in problem-solving
	Navigators organized in each department involved	Navigators organized in a centralized unit at hospital level	Navigators organized in each department involved	Navigators organized in a centralized unit at hospital level

**Table 3:** Variables influencing care coordination in the breast cancer pathway in the four hospitals, 2019 [56].

	University Hospital A	Community Hospital B	University Hospital C	Community Hospital D
No of patients diagnosed with cancer	588	223	412	66
Variation in monthly number of patients starting standardized CPP	Average: 69	Average: 35	Average: 77	Average: 14
	Max/Min: 84/52	Max/Min: 47/19	Max/Min: 113/40	Max/Min: 23/7
No of patients in standardized pathway receiving their first cancer treatment	S *: 389	S *: 157	S *: 249	S *: 48
	C *: 218	C *: 15	C *: 179	C *: 18
	Sum: 607	Sum: 172	Sum: 428	Sum: 66

* Surgery (S), Chemotherapy (C).

**Table 4:** Variables influencing care coordination in the colorectal cancer pathway in the four hospitals, 2019 [56].

	University Hospital A	Community Hospital B	University Hospital C	Community Hospital D
No of patients diagnosed with cancer	330	230	186	95
Variation in monthly number of patients starting standardized pathway	Average: 49	Average: 73	Average: 35	Average: 21
	Max/Min: 64/36	Max/Min: 91/60	Max/Min: 43/13	Max/Min: 29/12
No of patients in standardized pathway receiving their first cancer treatment	S *: 174	S *: 161	S *: 183	S *: 61
	C *: 34	C *: 15	C *: 75	C *: 11
	R *: 138	R *: 0	R *: 62	R *: 1
	Sum: 346	Sum: 176	Sum: 320	Sum: 73

* Surgery (S), Chemotherapy (C), Radiotherapy (R).

**Table 5:** Variables influencing care coordination in the ovarian cancer pathway in the four hospitals, 2019 [56].

	University Hospital A	Community Hospital B	University Hospital C	Community Hospital D
No diagnosed with cancer	251	27	46	9
Variation in monthly number of patients starting standardized pathway	Average: 14.6	Average: 4.9	Average: 6.2	Average: 1.1
	Max/Min: 21/12	Max/Min: 11/3	Max/Min: 11/3	Max/Min: 3/0
No of patients in standardized pathway receiving their first cancer treatment (surgery)	175	59	75	13

**Table 8:** Characteristics of organizational aspects related to the three diagnoses.

	Breast Cancer	Colorectal Cancer		Ovarian Cancer
		Colon	Rectum	
**Organization of** **referral and the** **diagnostic workup**	*Referrals to local hospitals by GPs, or after positive screening:* Initial diagnostic workup in sheltered environment managed by radiologists *Referrals to regional hospitals from local hospitals:* Specialized diagnostic imaging in sheltered environment	*Referrals to local and regional hospitals, from GPs or local hospitals:* Diagnostic workup in general gastrointestinal department with allocated slots for CPPs, otherwise non-sheltered environment		*Referrals from GPs,* *private providers and local hospitals:* Suspected or detected in non-sheltered gynecology or gastro-medicine department *Referrals to regional* *hospitals:* Sheltered gynecological cancer departments
**Participants in MDT meetings**	Radiologist, breast surgeon, pathologist and oncologist (except university hospital C)	Gastro surgeon, radiologist, oncologist, pathologist and, only in community hospital B, gastro-intestinal physician		Gynecologist, radiologist and pathologist
**Organization** **of treatment**	*Surgery:* Specialized surgeons. Primary reconstructive surgery performed cross-disciplinary with plastic surgeons *Regional breast cancer:* Initial oncological treatment demanding close dialogue between oncologist and surgeon	*Local colon* *cancer:* Surgery in community hospital also comprising other elective and acute diagnoses	*Local rectum cancer:* Surgery in community hospital also comprising other elective and acute diagnoses *Regional rectum* *cancer*: Possible cross-disciplinary surgery at regional hospital	Surgical and oncological treatment managed by specialized regional department for gynecological cancer
		*Metastases:* Cross-disciplinary decision process in MDTs and possibly synchronic surgery if detected simultaneously		
**Organization of state of remission follow-up**	*Surgery as primary treatment:* Follow-up by breast surgeon at local hospital *Neoadjuvant treatment:* Follow-up by oncologist at local hospital	*Surgery as first treatment:* Follow-up by gastro surgeon at local hospital *Neoadjuvant treatment:* Follow-up by oncologist at local hospital *Metastatic surgery or advanced* *rectum surgery at regional* *hospital:* Follow-up by the treating unit		First follow-up by gynecological cancer unit at regional hospital and subsequently at local hospital

## Data Availability

The data presented in this study are available on request from the corresponding author. The data are not publicly available due to privacy.
